# Imaging Characteristics of Pediatric Renal Cell Carcinoma and Wilms Tumor and Its Impact on Their Management and Outcomes—A Case Report and Review of Literature

**DOI:** 10.15586/jkc.v12i4.388

**Published:** 2025-10-28

**Authors:** Anand Chetan Shah, Prasanth Srinivasan, Shalini Shree Krishnamurthy, Shirley Sunder Singh, Venkatraman Radhakrishnan, Anand Raja

**Affiliations:** 1 Department of Surgical Oncology, Cancer Institute (W.I.A), Chennai, India;; 2 Department of Medical Oncology, Cancer Institute (W.I.A), Chennai, India;; 3 Department of Oncopathology, Cancer Institute (W.I.A), Chennai, India

**Keywords:** pediatric, PET, renal cell carcinoma, translocation, Wilms tumor

## Abstract

Renal cell carcinoma (RCC) in children is rare, comprising only 1.4–6.3% of pediatric renal tumors. Differentiating RCC from Wilms tumor, the most common pediatric renal tumor is crucial due to differing management and prognosis. Imaging characteristics, such as the presence of calcifications and cystic components, guided the decision to perform surgery without pretreatment biopsy, reducing the risk of needle tract seeding. Translocation-associated RCC is the most common subtype in children, and surgical resection remains the cornerstone of treatment. Long-term follow-up is essential due to the potential for late recurrences. We are reporting the details of a 4-year-old boy, who presented with a 15-day history of fever and abdominal distension, accompanied by a ballotable mass in the right lumbar region. Imaging studies, including a CT scan, revealed a large complex cystic mass in the right kidney, consistent with Bosniak category IV, and enlarged paraaortic nodes. Further evaluation with an FDG-PET scan confirmed the uptake only in the right kidney. The child underwent a right radical nephrectomy with retroperitoneal lymph node dissection. Histopathology revealed translocation-associated RCC features, characterized by slender papillae, psammoma bodies, necrosis, and uniform epithelial cells with hyperchromatic nuclei. Immunohistochemistry showed positivity for markers including TEF3, keratin, and vimentin, with a KI-67 proliferation index of 10–20%. The final stage was pT2aN0, and the patient had an uneventful recovery, with no recurrence at 36 months of follow-up.

## Introduction

Childhood renal tumors account for around 7% of all childhood cancers, the commonest being Wilms tumor in the first decade of life (1). Renal cell carcinoma (RCC) is more common than Wilms tumor in the second decade of life (1). Among RCC, translocation-associated RCC (20–40%) is commonest in the pediatric population (2). Factors influencing prognosis include symptomatic presentation, performance status, stage, grade, and histology (3). Surgery forms the mainstay of treatment and results in a cure when the tumor is localized and completely resected (4). We present the case of a child who reported to our institute with a renal mass and was ultimately diagnosed with RCC.

## Case Report

**Figure 1:**
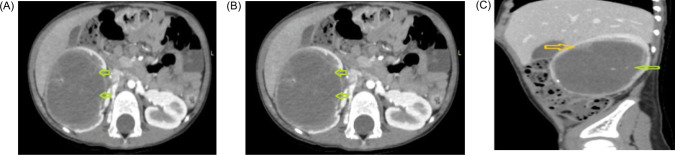
Contrast-enhanced CT scan (abdomen and pelvis). (A) Axial noncontrast images, showing a heterodense solid cystic mass in the right kidney, showing the presence of calcifications (yellow arrow). (B) and (C) Axial and sagittal CECT images showing a cystic lesion in the right kidney with thin enhancing internal septations (green arrow) and peripherally placed solid components (yellow arrow).

**Figure 2:**
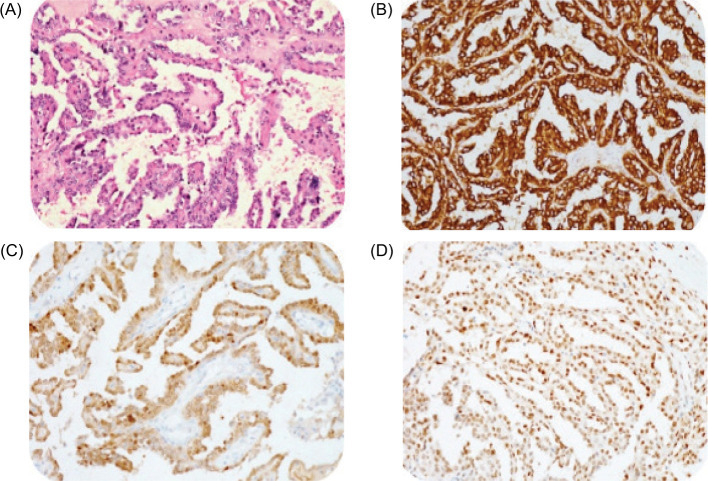
(A) Papillary structures lined by round to polygonal cells with moderate cytoplasm and vesicular nuclei with nucleoli. H&E 200X. (B) Tumor cells show diffuse positive reaction for cytokeratin. DAB 200X. (C) Tumor cells show a positive reaction for PAX8. DAB 200X. (D) Tumor cells show a positive reaction for TFE3. DAB 200X.

A 4-year-old boy presented to our institute with abdominal distension and a fever of 15 day duration. Clinical examination revealed a ballotable mass in the right lumbar region. Routine blood investigations including renal function tests (RFT) were within normal limits. 24-hour

urinary vanillylmandelic acid (VMA) level was 0.4 mg/day (normal: 0–8). A computerized tomography scan (CT) revealed a large single complex cyst of size 8.6 x 6.3 x 6.1cm in the right kidney with enhancing septations, peripheral wall calcifications, and ill-defined soft tissue component within the cyst wall (BOSNIAK category IV) with few enlarged para-aortic nodes (Figure 1). CT scan of the chest was negative for metastasis. The fludeoxyglucose-18 (FDG) positron emission tomography (PET) scan revealed uptake only in the right kidney with no other sites of abnormal metabolic uptake. He underwent a right radical nephrectomy with retroperitoneal lymph node dissection (paracaval, precaval, and interaortocaval). Light microscopy revealed slender papillae with hyalinized fibrovascular cores, papillae lined by a single layer of uniform-looking epithelial cells with hyperchromatic nuclei, psammoma bodies, and necrosis (70%). Mitosis was scanty. Immunohistochemistry (IHC) showed positive reaction to transcription factor E3 (TEF3), keratin, vimentin, cytokeratin (CK-7), cluster of differentiation (CD10), and carbonic anhydrase-9, and negative reaction to alpha-methylacyl coenzyme-A racemase (AMACR), Epithelial membrane antigen (EMA), and variable Paired box gene (PAX-8) (Figure 2). KI-67 was 10–20%. The final histopathology was suggestive of translocation-associated RCC. All nodes were free (0/40). He was staged as pT2aN0. The patient had an uneventful postoperative recovery period and continues to be on regular follow-up with a disease-free survival of 36 months.

## Discussion

Wilms tumor is the most common pediatric renal tumor in the first decade of life (85%) (5). Other common tumors include RCC (1.8–6.3%) (1), clear cell sarcoma, congenital mesoblastic nephroma, malignant rhabdoid tumors, and lymphoma (6). The peak incidence of Wilms tumor occurs around 3 years of age (5), while RCC presents between 9 and 15 years of age (7). RCC accounts for 1.4% of all renal tumors in patients younger than 4 years,15–20% in patients aged 5–9 years, and 52.6% in patients aged 10–15 years (5). Differentiating between RCC and Wilms tumor in children less than 5 years with renal masses is important as the management and prognosis of both conditions vary widely. Clinical, radiological, therapeutic, and prognostic differences between RCC and Wilms tumor are summarized in Table 1. Wilms tumor typically presents as a large, solid, cystic renal mass with infrequent calcifications and less common lymph nodal involvement, whereas RCC tends to be smaller, predominantly cystic, and more frequently associated with calcifications and regional lymphadenopathy (1).

**Table 1:** Clinical, radiological, and pathological differences between Wilms tumor and renal cell carcinoma.

Parameters		Wilms tumor	Renal cell carcinoma
Age		1–5 years (peak 3–4 years)	More than 5 years (predominately 2nd decade)
Clinical presentation	Local symptoms	Asymptomatic abdominal mass	Hematuria, abdominal pain and abdominal mass
	Metastasis	Less frequent presentation with bone metastasis	High chance of metastasis to bone
Radiology	Size	Large mass	Small mass
	Consistency	Solid but can be cystic	Predominately cystic (more than Wilms)
	Calcifications	Less frequent	More frequent
	Involvement of nodes	Less frequent	More frequent
Management		Multimodality (surgery and chemotherapy ± radiotherapy)	Surgery (role of chemo, radiotherapy and immunotherapy not clear)
Microscopically		Solid single mass	Hemorrhagic and necrotic areas with calcifications
Prognosis	Stage I	98.4%	92.4%
	Stage II	97.7%	84.6%
	Stage III	94.4%	72.7%
	Stage IV	86.1%	13.9%

Our patient, a 4-year-old boy presented with a fever and abdominal mass. Differentiation between Wilms tumor and RCC based on imaging criteria is of utmost importance at this age where RCC is rare. Diagnostic imaging revealed calcifications, cystic mass, and enlarged paraaortic nodes. RCC was more likely than Wilms tumor based on imaging characteristics of our index child.

*Performing a needle biopsy can clear the diagnostic dilemma*, but it comes with the attendant risk of needle tract seedling. This might be of little consequence in Wilms tumor where multimodal treatment would negate the risks of upstaging. This is more so in a case like ours with a large cystic component that can disseminate widely along the biopsy tract. Biopsy also has complications. Vujanic et al. (8) performed a biopsy for all pediatric renal tumors. Serious complications were reported in 182 patients (punch canal metastasis, massive bleeding, and death from tumor rupture).

Given the above considerations, we elected not to perform a biopsy and proceeded with radical nephrectomy with a presumptive diagnosis of RCC. Biopsy and histopathological confirmation is mandated for all patients with metastatic RCC before initiation of systemic therapy. This approach aligns with the treatment strategy for Wilms tumor, where biopsy and preoperative chemotherapy followed by delayed nephrectomy is often a justifiable and standard approach. Postoperative Histopathology was suggestive of translocation-associated RCC.

Renal cell carcinoma is different in adults and children. *Clear cell carcinoma is the most common in adults*. In the pediatric population, translocation-associated RCC is the commonest (35–50%) (9) followed by papillary RCC, clear cell RCC, and medullary RCC (1). Translocation-associated RCCs typically involve the microphthalmia-associated transcription factor (MiT) family. The most commonly observed fusion partners include transcription factor E3 (TFE3) and transcription factor EB (TFEB), with the corresponding genes located on chromosomes Xp11.2 and 6p21, respectively (1). Nonmetastatic RCC is treated through surgical resection. Light microscopy and IHC characteristics of different types of RCC are summarized in Table 2 (1, 9).

**Table 2:** Pathological features of renal cell carcinoma subtypes.

Translocation-associated RCC (Xp11.2)	Papillary or nested architecture with ample acidophilic cytoplasm IHC for TFE3 is confirmatory HMB-45 (Human melanoma black), CD10 are often expressed cytokeratins or EMA-negative
Papillary RCC (Type 1 and 2)	Single layer of cuboidal cells with scant cytoplasm (type 1) and high – nuclear-grade pseudostratified cells with eosinophilic cytoplasm (Type 2) CK7 is positive in 87% of (Type 1) and 20% of (Type 2) lesions EMA, Vimentin, and AMACR are typically positive in both types
Clear cell RCC	Clear cytoplasm and cells are arranged in nest with intervening blood vessels Diffusely positive for CK-7 and negative for CD10
Medullary RCC	High-grade epithelial cells with acidophilic cytoplasm arranged in a tubular, often cribriform architecture are occasionally solid or sarcomatoid. Characteristic microscopic features include desmoplasia and an acute inflammatory reaction Positive for Pancytokeratin AE1/AE3 and EMA negative for high molecular weight cytokeratin show nuclear accumulation of p53 protein.

The tumor stage appears to be the most important factor for survival (10). The overall survival of pediatric RCC is about 63%, with survival rates for Stages I–IV at 92.4, 84.6, 72.7, and 13.9%, respectively (11). Little is known about the outcome or the optimal treatment of the different subtypes of childhood RCC (9). Translocation RCC is associated with a risk of lymph nodal metastasis at diagnosis. Studies have reported variable outcomes, with some showing good prognosis despite lymph node metastasis while others showing poor outcomes regardless of the stage (9). Papillary RCC is associated with a disease limited to the kidney in a majority of cases (9). Renal medullary carcinomas typically present with distant metastasis at diagnosis, and survival seldom exceeds a year regardless of adjuvant therapy (12). Most recurrences and deaths in pediatric RCC usually occur within the first 2 years after diagnosis, although late recurrences are frequent (4).

## Conclusion

Pediatric renal tumors are not always Wilms tumor. Distinguishing clinical and radiological features enables an appropriate differential diagnosis and influences proper treatment. Pretreatment biopsy can be avoided in RCC based on imaging characteristics. Complete resection of localized disease improves survival. Strict long-term follow-up is required.

## Data sharing

The data that support the findings of this study are available from the corresponding author upon reasonable request.

All procedures performed in studies involving human participants were per the ethical standards of the institutional and/or national research committee and with the 1964 Helsinki Declaration and its later amendments or comparable ethical standards. Informed consent was obtained from the participant included in the study.

This research was exempt from the Institutional Review Board or Ethics Committee as it is an isolated case report and patient consent has been obtained.

The participant was informed about the purpose of the study, the procedures involved, and their right to withdraw at any time without any consequences.

The confidentiality and anonymity of the participant were ensured throughout the study.

## Author Contributions

Anand Chetan Shah: Conceptualization, Writing and editing; Prasanth Srinivasan: Conceptualization, Writing and editing; Shalini Shree Krishnamurthy: Conceptualization; Shirley Sunder Singh: Resources and Supervision; Venkatraman Radhakrishnan: Resources and Supervision; Anand Raja: Resources and Supervision, Final approval.
